# Management of a giant retroperitoneal leiomyoma: a case report

**DOI:** 10.1186/s13256-018-1617-z

**Published:** 2018-03-26

**Authors:** O. Karray, R. Boulma, A. Abdi, A. Ben Miled, A. Dhaoui, N. Menif, K. Bellil, H. Khouni, A. Chouchen

**Affiliations:** 1 Urology Unit, Interior Security Forces Hospital, La Marsa, Tunisia; 2 General Surgery Department, Interior Security Forces Hospital, La Marsa, Tunisia; 3 Radiology Department, Charles Nicolle Hospital, Tunis, Tunisia; 4 Pathology Department, Interior Security Forces Hospital, La Marsa, Tunisia

**Keywords:** Leiomyoma, Retroperitoneal neoplasms, Embolization, Surgery

## Abstract

**Background:**

Leiomyomas are benign tumors observed mainly in adult women. The retroperitoneum is a rare location for leiomyomas; almost 100 cases have been reported. Because retroperitoneal leiomyomas are paucisymptomatic and the tumor size at diagnosis is relatively large, surgical management is challenging. Regular follow-up is required because recurrence and malignant sarcomatous transformation have been described in a few cases.

**Case presentation:**

We report a case of a 52-year-old North African woman with a 22-cm retroperitoneal leiomyoma. A preoperative embolization was performed 2 days before surgery. The clinical, therapeutic, and evolutive aspects of this rare entity are discussed.

**Conclusions:**

Despite its benignity, retroperitoneal leiomyoma is a challenging diagnostic, therapeutic, and evolutive condition. Surgeons must consider mainly the tumor’s vascularization. Regular follow-up is mandatory because malignant transformation cannot be excluded.

## Background

Retroperitoneal leiomyoma is a rare and benign condition. It is infrequently observed among primary retroperitoneal neoplasms. The diagnostic approach is challenging because retroperitoneal leiomyomas may be confused with renal tumors. Tumor vascularization must be defined and considered before surgery because it usually arises from the aorta and the renal artery. In this report, we describe a case of a patient with a retroperitoneal leiomyoma requiring preoperative arterial embolization. The diagnostic, therapeutic, and evolutive aspects are discussed.

## Case presentation

A 52-year-old North African woman consulted our department with a surgical emergency for recent left flank pain associated with a palpable tender solid mass. The patient is a married mother of three children and working as a secretary in public administration. She did not have any past medical history. She delivered two of her children by cesarean section. She complained of chronic constipation of 6 months’ duration. She was apyretic and did not complain of vomiting or recent disturbance in bowel motility. She had no urinary or gynecological disorders. She did not present with sensory or motor focal deficits or visual or speech disorders. The palpable mass was located in the left flank and the iliac fossa, with a posterior dorsal extension and regular margins. Deep palpation of the mass caused tenderness. The patient had no jaundice, and her urine and feces were not discolored.

The patient’s blood test results were normal. Her liver and kidney function was sufficient. Her white blood cell count was 7000/mm^3^. Her hemoglobin level was 13.5 g/dl. Her platelet count was normal. Her C-reactive protein concentration was 11 mg/L. Her CA-125 and carcinoembryonic antigen levels were within normal range.

Abdominal ultrasonography revealed a voluminous left-sided heterogeneous tissue abdominopelvic mass with a mass effect on the left kidney. Doppler imaging showed that the tumor was nonvascularized.

**Fig. 1:**
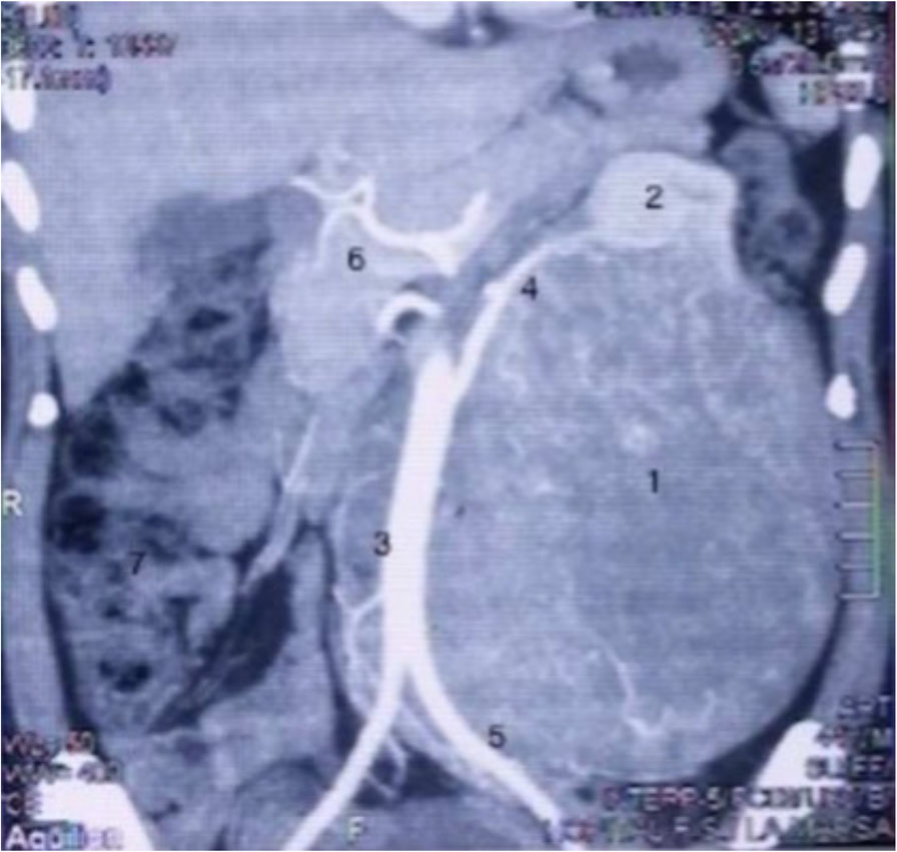
A retroperitoneal tumor with a mass effect on the left kidney, the aorta, and the bowel. 1 = Tumor, 2 = left kidney, 3 = aorta, 4 = left kidney artery, 5 = iliac artery, 6 = pancreas

On an abdominopelvic computed tomographic (CT) scan, the mass measured 16 × 17 × 22 cm. It was well-limited, heterogeneous, and hypervascularized. The vascularization seemed to arise exclusively from the renal artery. The mass effect involved the left kidney and its hilum, the left ureter, the left colon, and the inferior mesenteric vascular axis. The renal vein and the inferior vena cava were permeable (Fig. 1).

**Fig. 2:**
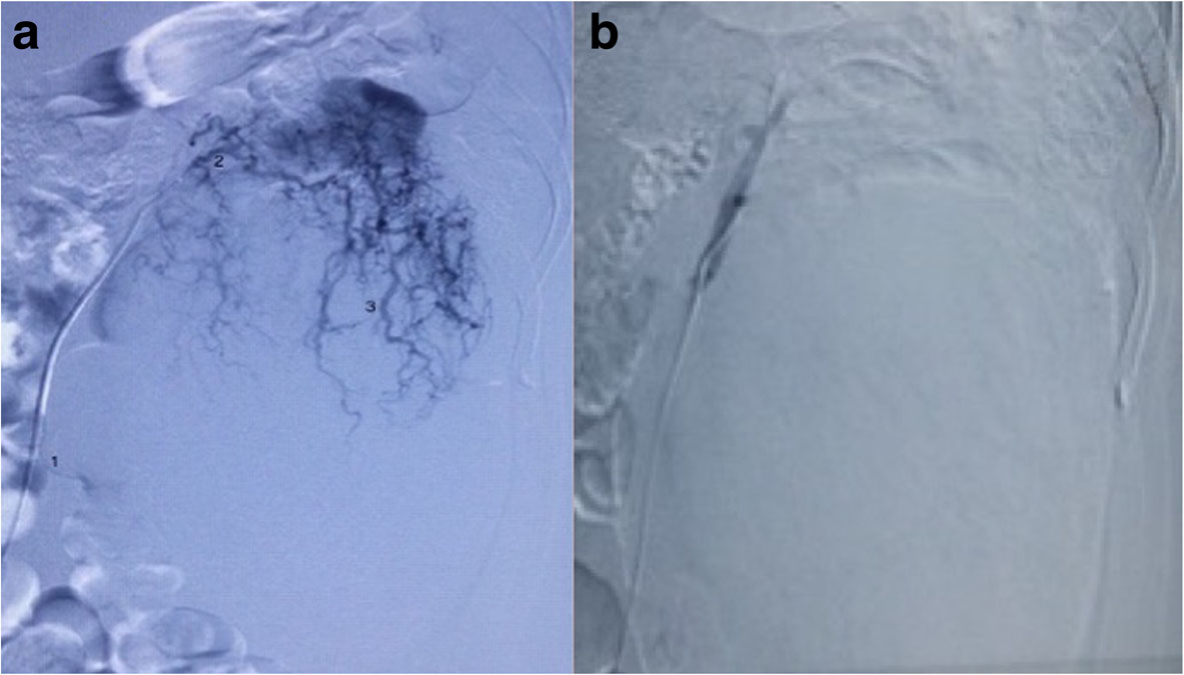
**a** Abundant and anarchic vascularization of the tumor, provided exclusively from the left renal artery. **b** Embolization of the renal artery led to complete disappearance of the tumor vascularization

Angiography confirmed the CT findings because the tumor vessels emerged exclusively from the renal artery. Embolization of the renal artery was performed using a transfemoral approach, which led to total disappearance of the tumor vascularization (Fig. 2). The patient had no fever or systemic inflammatory signs after embolization.

**Fig. 3:**
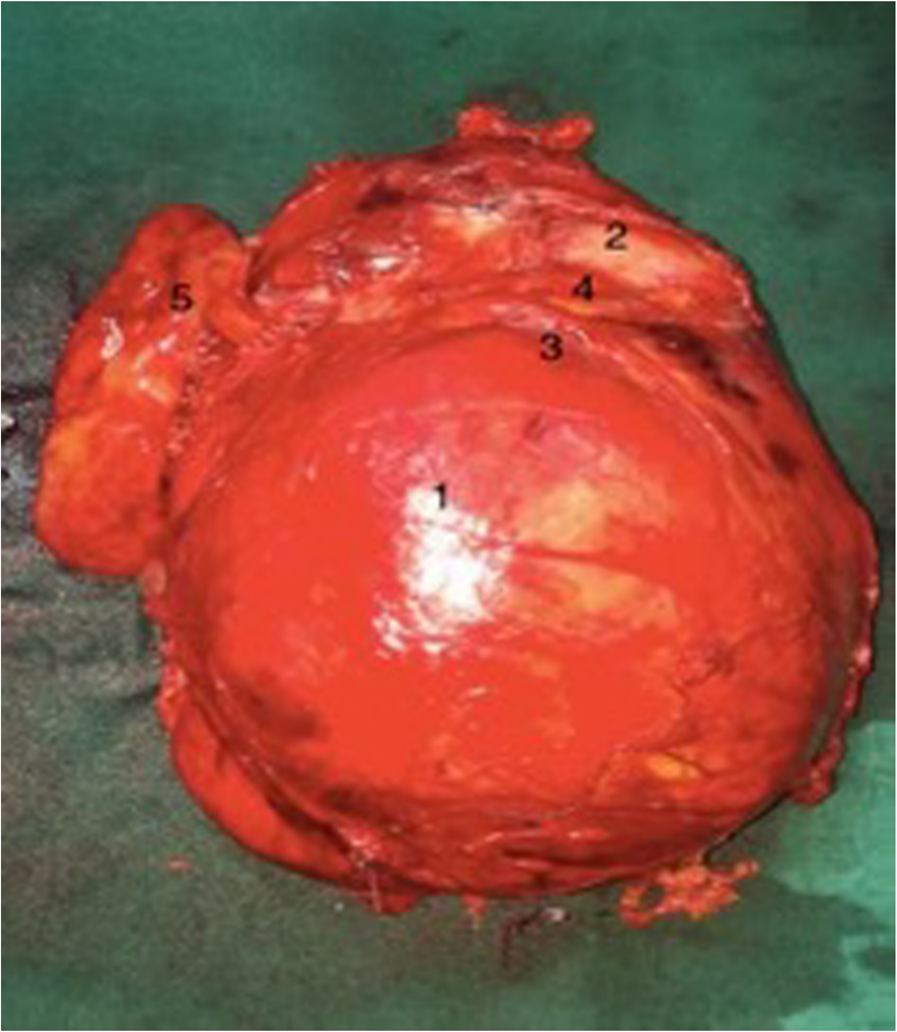
Operative specimen. 1 = Tumor, 2 = left kidney, 3 = tumor capsule, 4 = left kidney capsule, 5 = the kidney’s hilum

After 2 days of opioid analgesia, the patient underwent surgery. A midline incision allowed exploration of the abdominal cavity. The results of the operative exploration were similar to the scan findings. The left kidney was pushed to the median line. The mass seemed to be firmly adherent to the kidney and its hilum. The dissection plane was not obviously identifiable. The tumor had a smaller size after embolization, but it was not dissociable from the kidney. Its manipulation was not particularly hemorrhagic. The first step was the identification of the ureter and clamping of the renal artery and the vein. A thorough dissection of the tumor from adjacent structures was performed, allowing a one-piece resection of the mass with the left kidney (Fig. 3).

The patient’s postoperative course was uneventful. She was discharged on the fourth postoperative day.

**Fig. 4:**
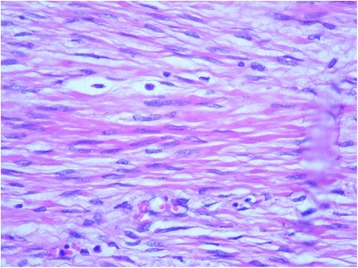
Histological specimen showing mesenchymal proliferation and containing smooth muscle cells in a hypervascularized stroma

**Fig. 5:**
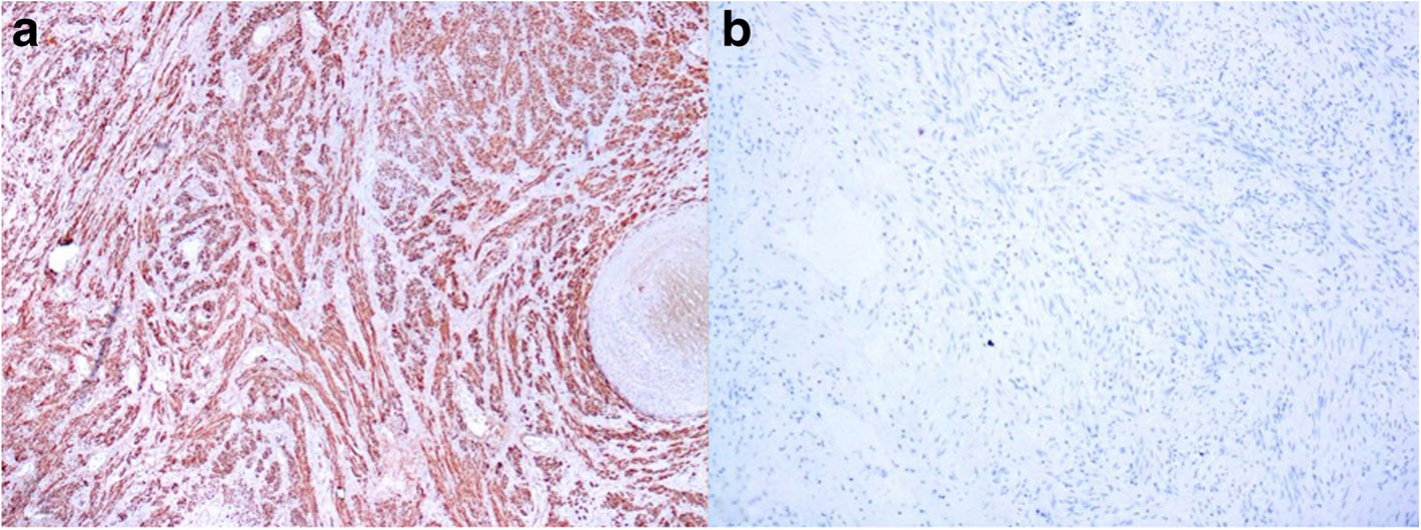
Immunohistochemical analysis revealed reactions that were (**a**) positive for desmin and (**b**) negative for HMB-45

Histological examination of the tumor demonstrated it to be a benign mesenchymal proliferation involving regular smooth muscle cells grown in a hypervascularized stroma. Focal hyalinized spots were observed. The mitotic index was not high. No fat cells or necrotic lesions were present (Fig. 4). The tumor proliferation did not involve the renal parenchyma. The results of immunohistochemical analysis were negative for HMB-45, CD117, DOG1, PS100, and CD34. The finding for the Ki-67 proliferation index was also negative. Tumor cells were positive for desmin, caldesmon, and actin, confirming the diagnosis of retroperitoneal leiomyoma (Fig. 5). The kidney and its capsule were free from any histological neoplastic lesions.

The patient was followed regularly for 2 years. The results of her clinical examination were normal. Her renal function was conserved. An abdominal CT scan ruled out any local or distant recurrence.

## Discussion

We report a new case of a 52-year-old woman with a retroperitoneal leiomyoma. The tumor measured 22 cm, and the CT scan aspects were confused with a voluminous renal tumor. Vascularization was exclusively from the renal artery, which is rarely observed in retroperitoneal leiomyomas. Preoperative embolization of the renal artery 2 days before surgery was performed safely and allowed decrease of the tumor’s size. The tumor was not dissociable from the kidney, and the operative specimen included the retroperitoneal mass and the kidney in one piece. Immunohistochemical markers were distinctive to ensure diagnostic accuracy.

Leiomyomas are developed from smooth muscle cells and are rarely located in the retroperitoneum. To the best of our knowledge, around a hundred of cases have been described in the English literature; 40 of them contain sufficient data for study [1].

Retroperitoneal smooth muscle cell tumors are often diagnosed fortuitously, and symptoms are related to compression of adjacent structures. The mean size at diagnosis is 12 cm [2]. Eighty percent of them are malignant, represented by leiomyosarcomas [3]. Predicting malignancy of smooth muscle tumors relies on histomorphological evidence, including the tumor size, the margin’s infiltrating character, the important mitotic activity index, and cytological atypia. These criteria may differ, depending on the tumor’s location. Retroperitoneal leiomyomas in female patients may express significant mitotic activity index [4].

The pathogenesis of retroperitoneal leiomyomas is still unclear. The role of gonadotropic hormones seems to be influential. The uterine location, the one most often described, occurs mainly during the genital activity period. Up to 40% of retroperitoneal cases are associated with synchronous or previously operated uterine myomas [1]. This theory is particularly plausible because only nine cases have been reported in men [5], and the density of estrogen and mainly of progesterone receptors is remarkably important [6]. Stutterecker *et al*. described the eventuality of the development of embryonic vessels in remnant musculature. This hypothesis is supported by the previous case reports describing pulmonary and heart locations [7].

The diagnosis is rarely established preoperatively [1]. Symptoms are not specific and are related to the mass effect of the tumors [8]. It is important to mention that most retroperitoneal leiomyomas are independent from the uterus in the pelvic floor [1]. They develop in the upper part of the retroperitoneum in 30% of cases [9]. In four patients, the leiomyomas were reported to be in the anterior retroperitoneum, in the Retzius space, or in the adnexa of uterus [5, 10].

Ultrasound and CT scan aspects are not evocative. The tumor is usually described as a voluminous, heterogeneous, hypervascularized mass [4]. Preoperative imagery is mainly of interest to define the anatomical limits of the tumor precisely. Magnetic resonance imaging may be useful to differentiate leiomyoma from leiomyosarcoma, such as in the case of uterine myomas [11].

Differential diagnoses concern other spindle cell tumors, such as leiomyosarcomas and stromal tumors. Other rare diagnoses, such as malignant peripheral nerve sheath tumor, inflammatory myofibroblastic tumor, and pleomorphic sarcoma, have been described [4]. Immunohistochemical analysis is of primary importance in this context. The most essential markers, ensuring accurate differentiation between leiomyomatous and stromal tumors particularly, are desmin, CD34, and CD117 [12].

Tumor biomarkers, especially the CA-125 and carcinoembryonic antigens, may be elevated in patients with huge tumors. Preoperative documented elevation of these antigens can be helpful in further follow-up [13].

Surgery is the only curative option. Open surgery was the option chosen in most of the previously reported retroperitoneal leiomyoma cases. A laparoscopic approach was described in only two cases [1]. The difficulty with laparoscopic or robot-assisted approaches is related mainly to the relatively large tumor size at diagnosis and the adherence to adjacent structures [14]. The principal aim is to ensure a one-piece excision of the tumor and to conserve the integrity of the surrounding organs and large vessels [15]. Once benignity is confirmed histologically, recurrence and malignant transformation are extremely rare [1]. Nevertheless, the few reported cases justify a thorough clinical and radiological follow-up. Other therapeutic options can be discussed on an individual basis. A luteinizing hormone-releasing hormone analogue has been proposed, despite the lack of efficacy and the recurrence of evolutive symptoms upon discontinuation of treatment [16]. Arterial embolization was also described as a preoperative option in patients with huge retroperitoneal tumors. It is useful mainly to reduce operative hemorrhagic incidents and to slightly reduce the tumor size [17]. Surgery should be performed within 2 days, and analgesia must be strengthened because the pain is usually intense [5]. Even though some incidents have been described, such as arterial dissection or systemic embolization, tumor artery embolization is usually a safe technique to facilitate the surgical approach in patients with voluminous or symptomatic retroperitoneal tumors [16]. In our patient, the tumor was totally vascularized from the renal artery, whereas most of the described embolization procedures of the retroperitoneal tumors concerned lumbar arteries and secondarily the renal artery. The presence of a lumbar artery allows sparing of the renal artery from embolization when preoperative imaging confirms the retroperitoneal and extrarenal location of the tumor. Thus, nephrectomy can be avoided.

## Conclusions

Retroperitoneal leiomyoma is a challenging diagnostic and therapeutic situation. Histological diagnosis is usually postoperative because radiological features are not conclusive of benignity. Surgeons must consider the vascularization of the tumor because it may arise from the renal artery in some cases. Thorough clinical and radiological follow-up is required because of the potential malignant transformation.
